# Distortion Correction in EPI Using an Extended PSF Method with a Reversed Phase Gradient Approach

**DOI:** 10.1371/journal.pone.0116320

**Published:** 2015-02-23

**Authors:** Myung-Ho In, Oleg Posnansky, Erik B. Beall, Mark J. Lowe, Oliver Speck

**Affiliations:** 1 Department of Biomedical Magnetic Resonance, Institute for Experimental Physics, Otto-von-Guericke University Magdeburg, Magdeburg, Germany; 2 Department of Radiology, Imaging Institute, Cleveland Clinic, Cleveland, Ohio, United States of America; 3 German Centre for Neurodegenerative Diseases (DZNE), Site Magdeburg, Magdeburg, Germany; 4 Leibniz Institute for Neurobiology, Magdeburg, Germany; National Taiwan University, TAIWAN

## Abstract

In echo-planar imaging (EPI), such as commonly used for functional MRI (fMRI) and diffusion-tensor imaging (DTI), compressed distortion is a more difficult challenge than local stretching as spatial information can be lost in strongly compressed areas. In addition, the effects are more severe at ultra-high field (UHF) such as 7T due to increased field inhomogeneity. To resolve this problem, two EPIs with opposite phase-encoding (PE) polarity were acquired and combined after distortion correction. For distortion correction, a point spread function (PSF) mapping method was chosen due to its high correction accuracy and extended to perform distortion correction of both EPIs with opposite PE polarity thus reducing the PSF reference scan time. Because the amount of spatial information differs between the opposite PE datasets, the method was further extended to incorporate a weighted combination of the two distortion-corrected images to maximize the spatial information content of a final corrected image. The correction accuracy of the proposed method was evaluated in distortion-corrected data using both forward and reverse phase-encoded PSF reference data and compared with the reversed gradient approaches suggested previously. Further we demonstrate that the extended PSF method with an improved weighted combination can recover local distortions and spatial information loss and be applied successfully not only to spin-echo EPI, but also to gradient-echo EPIs acquired with both PE directions to perform geometrically accurate image reconstruction.

## Introduction

Single-shot echo planar imaging (EPI) [1] is the most popular sequence in many neuroscientific and clinical studies that apply functional MRI (fMRI) or diffusion tensor imaging (DTI). A major shortcoming of EPI is geometric and intensity distortion in the phase-encoding (PE) direction due to its low effective bandwidth per pixel. The effects can be easily seen in regions near tissue/bone or tissue/air interfaces for human brain imaging. Due to increased geometric distortions up to 3 cm, especially at ultra-high field (UHF) such as 7T [2], rigorous corrections should be performed to accomplish reliable alignment with an anatomical reference image. Such correction is necessary for anatomically faithful EPI data analysis and interpretation.

Geometric distortions, appearing locally either as stretched or compressed pixels, alter the local spatial resolution in the acquired image. Locally increased spatial resolution is acquired in stretched regions, while locally decreased spatial resolution is obtained in compressed regions. Particularly in strongly compressed regions, where several pixels of the nominal acquisition grid are compressed into a single pixel, spatial information is lost. If one assumed a smoothly varying field inhomogeneity, it is possible to recover some of the information, however this assumption is violated in strongly warped regions and in these cases no unique solution exists [3]. Therefore, loss of spatial information cannot be fully recovered using the shift (or distortion) map alone with magnitude image-based resampling.

As an alternative to resolve the above problem, reversed phase gradient approaches [4,5,6,7,8] have been suggested. In these approaches, the same slice is acquired twice with EPI using opposite PE polarities, resulting in forward (PE blip-up) and reverse (PE blip-down) *k*-space trajectories. These approaches are based on the assumption that EPI acquisition with opposite PE polarity yields identical intensity, but opposite geometric distortion along the PE direction in the acquired image. Since the lost spatial information due to compression is retained in the corresponding EPI image with opposite distortions, a final image without loss of spatial information can be generated by combining the EPI pair after distortion correction. However, strong image compression leads to strong signal dropouts in gradient-echo EPI (GE-EPI) rather than signal summation of compressed voxels as in spin-echo EPI (SE-EPI) and these approaches [4,5,6,7,8] generally work on SE-EPIs with opposite distortions. In addition, accurate calculation of distortion information even from the SE-EPI pair can be erroneous due to lack of a unique solution between corresponding locations, especially in regions with strong field inhomogeneity. Furthermore, severely compressed regions should be less accounted for in the combination process since spatial information is already lost. In order to generate a distortion-free EPI image, therefore, there are still challenges in terms of how to correct distortions more precisely and how to combine the two images.

Point spread function (PSF) mapping allows very reliable distortion mapping due to its robustness to noise and field inhomogeneity [9]. In this method, an additional spin-warp PE gradient, which encodes the same spatial information as the EPI PE gradient, is added to the original EPI sequence. After 3D inverse fast Fourier transformation, the reconstructed PSFs are represented along the correlation (or diagonal) line in the spin-warp (or non-distorted) and EPI (or distorted) PE coordinates. Any distortion in the image is reflected in a deviation of the PSFs from the diagonal line along the EPI PE coordinates versus the spin-warp PE coordinates, and thus this deviation of the PSFs allows the accurate calculation of distortions. It has been suggested that a single delta approximation of the PSF can be used to extract distortion information as shift maps in the spin-warp [9,10,11], EPI [12], or both PE coordinates [13], but this resulted in a loss of information about distortion, and decreased the accuracy of correction. Recently, a newer study [14] revealed that a kernel for distortion correction can be obtained directly from the full PSF and thereby significantly improve the fidelity of EPI distortion correction, especially at UHF.

Here we propose an extension of the highly accurate PSF mapping method suggested by In and Speck [14] to resolve the remaining loss of spatial information in compressed regions. Therefore, a method for distortion correction of both forward- and reverse-PE EPI images is proposed that is based on a single PSF dataset acquired with only one PE direction. With current methods, the PSF reference scan must be performed with the same EPI PE polarity as the EPI thus requiring two PSF reference scans for the reverse gradient approach. Based on the hypothesis that the deviation of the PSFs will be mirrored along the distorted coordinates at the diagonal line if the PSF image is obtained with the reversed PE gradient, the proposed method extends the processing of one PSF reference dataset to result in distortion information for both PE polarities. In order to efficiently preserve spatial information from both distortion-corrected EPI images, we also investigated a suitably weighted summation to generate a distortion-free image with efficient combination of spatial information. Further we demonstrate that the extended PSF method can be applied not only to SE-EPIs, but also to GE-EPIs acquired with both PE directions to perform geometrically accurate image reconstruction. The methods are tested in phantoms and human subjects and compared to corrections with additional reference scans with identical PE polarity as well as with the reversed gradient approaches [4,5,6,7,8] suggested previously.

## Methods

### An extension of the PSF method for the reversed gradient approach

For simplicity, we consider the case of a single delta approximation of the PSF. Since the reconstructed PSF is obtained by multiplying proton density *ρ*(*s*) with the point spread function *H*(*y,s*), a single delta approximation of the magnitude PSF leads to [9,10,11]:

I(y,s)=ρ(s)H(y,s)≈ρ(s)δ(s+Δ(s)−y) (1)

where *δ* is the Dirac delta function, and *s* and *y* correspond to the spin-warp (non-distorted) and EPI (distorted) PE coordinates, respectively. Because the field inhomogeneity term Δ(*s*) is a function of *s*-coordinates, the PSF deviates from the diagonal line along the *y*-direction in the *s*-axis. Thus integration of the PSF data along the *y*- and *s*-directions yields the non-distorted and distorted reference (Ref.) images, respectively [9,10,11]:

I(s)=∫I(y,s)dy (2)

I(y)=∫I(y,s)ds (3)

If the PSF image *I*(*y,s*) is considered as the forward phase-encoded PSF image, *I*_*f*_(*y,s*), (hereafter referred to as the forward PSF), the PSF will be mirrored with -Δ(*s*) along the *y*-direction in the *s*-coordinates from the diagonal line in the reverse phase-encoded PSF image *I*_*r*_(*y,s*) (hereafter referred to as the reverse PSF) having opposite geometric distortions:

$$I_{r}(y,s)\approx \rho (s)\delta (s-\Delta (s)-y).$$ (4)

**Fig 1:**
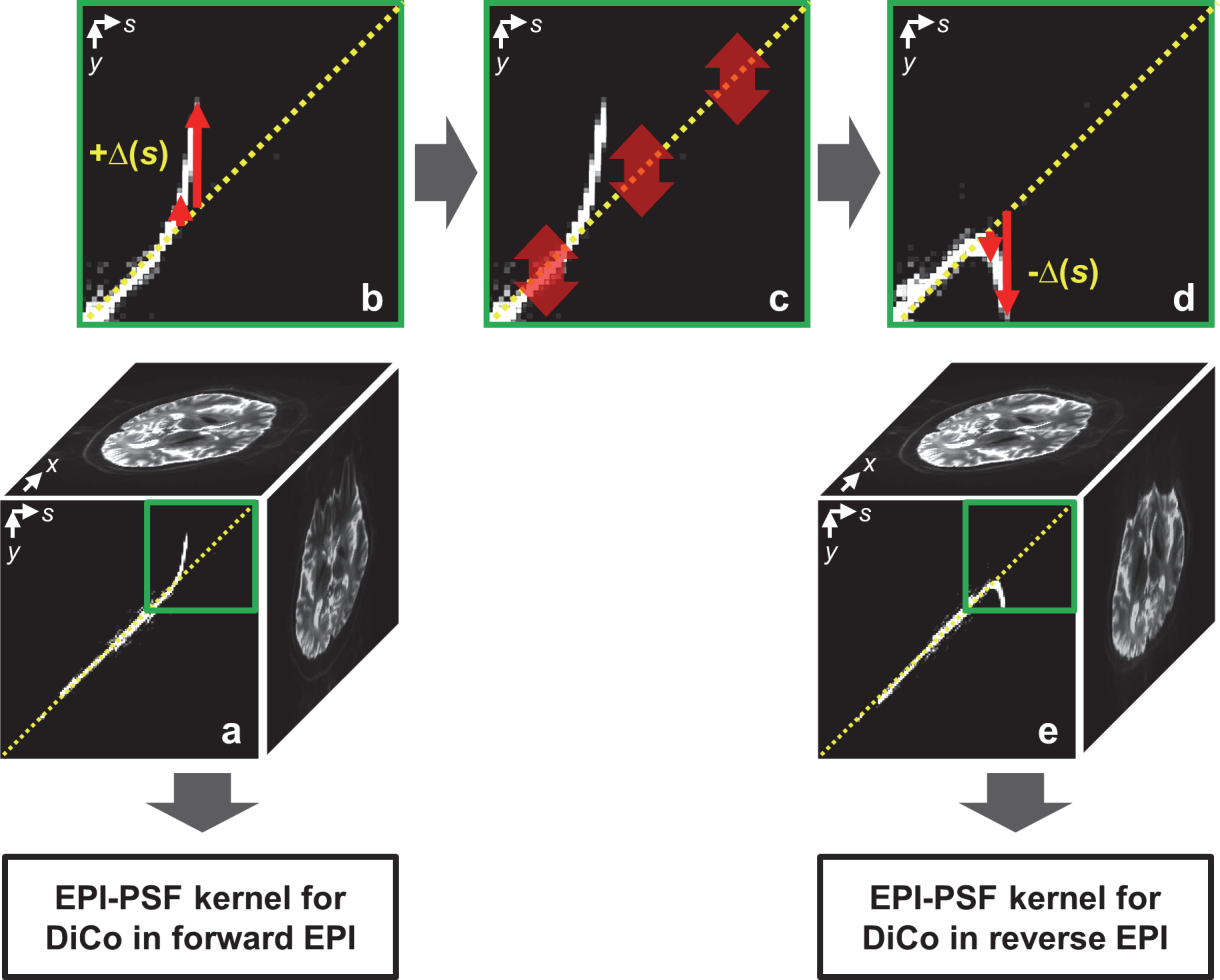
A diagram of extension of the PSF data from a single (for example, forward) PSF reference data: (a) measured forward 3D PSF data set, distortion-free (top) and distorted (right) reference images calculated respectively by Eqs. 2 and 3, (front) 2D PSF image in *y-s* plane at selected fixed *x*; (b) an expansion image from a small portion of the 2D PSF image in (a); (c) procedure of inversion of the 2D PSF image along the *y*-direction in the *s*-coordinates with respect to the diagonal line; (d) mirrored 2D PSF image; (e) an extended PSF data corresponding to the reverse PSF data. Finally, two EPI-PSF kernels for distortion correction (DiCo) in the both EPIs are calculated from the forward and extended PSF data.

It is clear that we can use the same assumption without restriction to a single delta approximation of the PSF. This idea is illustrated in the diagram of Fig. 1. A forward PSF data set (Fig. 1A) is obtained by a PSF reference scan after 3D inverse FFT. The deviation Δ(*s*) from the diagonal line due to the field inhomogeneity is shown in Fig. 1B. In order to yield an additional PSF image corresponding to the reverse PSF image *I*_*r*_(*y,s*) (Fig. 1E), the *y*-positions of the forward PSF image *I*_*f*_(*y,s*) are inverted with respect to the diagonal line (Fig. 1C) (hereafter referred to as the extended PSF). Therefore, two distortion correction kernels [14] for the forward and reverse EPIs can be calculated from the forward and extended PSF images, respectively. The proposed extension allows distortion correction for the forward and reverse EPIs without additional scan time required for the corresponding reverse PSF scan.

### EPI-PSF-based weighting maps for summation of the EPI pair

The PSF *H*(*y,s*), which represents the profile along the EPI PE direction (*y*) in the spin-warp PE coordinates (*s*) in the 3D PSF space is the system function for generating image distortions, as shown in Eqs. 1 and 3, and cannot be applied for distortion correction directly. Instead, a different PSF derived from Eq. 3, which corresponds to the normalized profile of the PSF along the spin-warp PE direction in the EPI PE coordinates (EPI-PSF), is used as a kernel for direct distortion correction [14]. From Eq. 9 in the reference [14], a distortion-corrected EPI image *I*_*c*_(*s*) can be written as:

$$I_{c}(s)=\int I(y)H_{c}(y,s)=\int I_{c}(y,s)dy.$$ (5)

This is a convolution product of a distorted EPI *I*(*y*) and the EPI-PSF kernel *H*_*c*_(*y,s*). After distortion correction by the EPI-PSF kernel, the signal will be increased in previously stretched regions due to the amount of image compression and vice versa in the regions with compressed distortions. Since the scale is changed by geometric distortion correction using the EPI-PSF kernel, it includes a modulation function to correct the signal intensity. An integration of the EPI-PSF kernel along the *y*-coordinates yields a modulation function as a weighting map *W*(*s*) in this study:

$$W(s)=\int H_{c}(y,s)dy.$$ (6)

The value in the weighting map *W*(*s*) departs from one when the EPI is compressed (higher than 1) or stretched (lower than 1) during the correction. Since we assume that the spatial information in the regions that were originally stretched is more reliable than the information in the compressed regions, the spatial accuracy of the distortion-corrected EPIs correlates with the values in the weighting map. Therefore, instead of simple averaging of the distortion-corrected forward *I*_*c,f*_(*s*) and reverse *I*_*c,r*_(*s*) EPIs, a weighted combination (WC) is proposed in this study in order to generate a combined distortion-free image *I*_*WC*_(*s*). This accounts for the amount of information present in each image, which preserves spatial accuracy:

$$I_{WC}(s)=\frac{W_{f}{\left(s\right)}^{n}I_{c,f}(s)+W_{r}{\left(s\right)}^{n}I_{c,r}(s)}{W_{f}{\left(s\right)}^{n}+W_{r}{\left(s\right)}^{n}},$$ (7)

where *W*_*f*_(*s*) and *W*_*r*_(*s*) correspond to the forward and reverse weighting maps according to Eq. 6 with the weighting exponent *n*. Note that it becomes a simple average when *n* is equal to zero.

### Experiments and post-processing

All phantom and *in-vivo* scans were performed on a 7T whole body scanner (Siemens Healthcare, Erlangen, Germany) using a 32 channel phase-array head coil (Nova Medical, Wilmington MA, USA). Single-shot EPI and corresponding PSF sequences developed by Zaitsev et al. [11] were modified to acquire paired EPI images with opposite PE polarity as separate, subsequent volumes. Thus for an EPI acquisition, all slices of a single given volume are acquired with forward PE followed by all slices of the same volume with reverse-PE. The Stejskal-Tanner diffusion encoding scheme was implemented to minimize echo-time (TE) at UHF and acquire diffusion-weighted images with high signal to noise ratio (SNR). The proposed method was applied to a home-made geometry phantom (designed with inner structure and filled with agar gel) to assess the correction fidelity without head motion or physiological confounds. Measurements in three healthy volunteers after institutional review board (Otto-von-Guericke University Magdeburg, Germany) approved written consent were performed to further evaluate the proposed method.

(I) First, we tested the hypothesis that distortion correction with the extended PSF is comparable to correction with the measured reverse PE PSF dataset. For this purpose pairs of SE-EPI and corresponding PSF data with opposite PE polarity were acquired. In phantom and *in-vivo* experiments, the imaging parameters for SE-EPI and -PSF scans were: 12 axial slices, slice thickness = 1.2 mm, TR/TE = 2000/54 ms, bandwidth BW = 1543 Hz/pixel, field of view (FOV) = 224 mm^2^, matrix size = 180^2^, partial Fourier 6/8, standard in-plane 2D GRAPPA factor = 3, in-plane 2D GRAPPA reference lines = 45. GRAPPA acceleration in the PSF dimension [11] was not applied in this study. For the PSF reference acquisition, an acceleration by a factor of 6 (corresponding to 30 EPI repetitions, acquisition time (TA) = TR×repetitions = 1 min.) was achieved by reduction of the field of view (rFOV) in the spin-warp PE dimension [11]. Total TA for pairs of EPIs and PSF data in this experiment was about 2 minutes for each phantom and *in-vivo* experiment.

The EPI-PSF kernel for distortion correction, distorted and non-distorted reference images were calculated as described in In and Speck [14]. The cross correlation between the EPI-PSF profiles in the forward and reverse PSF spaces was calculated along the *s*-axis. The maximum value (equal to 1) is reached only when identical EPI-PSF profiles at the same position are measured in both PSF spaces. This was performed with the forward and extended reverse PSF data due to the opposite distortions. A pair of EPI-PSF kernels, which is applied to the corresponding pair of EPIs for distortion correction, was obtained by the proposed method from each of the forward and reverse PE PSF reference data and two pairs of distortion-corrected images were obtained using the two PSF data. To fully cover the complete EPI-PSF profile for distortion correction, a kernel size of 13 was used in this study. Finally, in order to validate the correction quality, the distortion-corrected images were subtracted from each other.

(II) To demonstrate that accurate distortion correction with the proposed method is available even in GE-EPI, a pair of GE-EPIs with opposite PE polarity and forward phase-encoded GE-PSF data were acquired with identical protocols to the first experiment with the exception of TR/TE = 2000/22 ms. For the PSF reference acquisition, an acceleration factor of 4 (corresponding to 45 EPI repetitions, acquisition time (TA) = 1 min. 30 sec.) was used to obtain a distortion-free GE-reference (GE-ref.) image. Total TA for this experiment was about 2 minutes.

The forward and extended EPI-PSF kernels were obtained from the forward PSF reference data to correct distortions in the forward and reverse GE-EPIs. To verify further accelerations of the PSF scan, a non-equidistant PSF acquisition scheme (with a further FOV reduction of 2 plus 4 additional center PSF samples) [14] was applied, which requires only 25 repetitions for calculation of the kernel. A distortion-free image was finally generated by weighted combination of the two distortion-corrected images using the proposed weighting factors calculated from the EPI-PSF pair according to Eq. 6.

(III) *In-vivo* DTI experiments were performed to evaluate the robustness of the proposed method in human data. A SE-PSF dataset with forward PE gradient and pairs of diffusion weighted EPI (DW-EPI) with opposite PE polarity were acquired, corresponding to two averages in regular acquisitions. The imaging protocols were identical to the first experiment with the exception of TR/TE = 8200/58 ms and the use of 80 axial slices to cover the entire brain. The SE-PSF reference scan was performed with an acceleration factor of 3 (corresponding to 60 EPI repetitions, TA = 8 min.) [11] to yield a distortion-free SE-reference (SE-ref.) image and the number of diffusion directions for DW-EPI with each PE polarity was 30 and 12 (*b*-value = 1000 s/mm^2^) respectively with one and four averages. For this experiment, total TAs were 17 and 21 minutes, respectively.

To calculate the EPI-PSF kernels, only 25 PSF-encoding steps were used (with a further FOV reduction of 3 plus 5 additional center PSF samples) [14]. Based on the distorted (forward and reverse), distortion-corrected (forward and reverse), and combined DW-EPIs, five fractional anisotropy (FA) maps were calculated and compared in order to evaluate the spatial accuracy. Note that all distortion correction schemes in DW-EPIs were applied after eddy-current correction and registration to SE-EPI with *b*-value = 0 s/mm^2^. The FA map calculation, eddy-current correction, and registration were carried out in FSL (http://fsl.fmrib.ox.ac.uk/fsl/). Additionally, a suitable value of weighting exponent *n* for combination was investigated to efficiently preserve the spatial information in the final FA map. For this purpose, mean differences *Diff*_*WC,n*_ between final FA maps with increasing weighting exponent of *FA*_*WC,n*_(*s*) and *FA*_*WC,n+1*_(*s*) were calculated:

$$Diff_{WC,n}=\frac{1}{N}\sum_{s=1}^{N}\left|\frac{FA_{WC,n+1}(s)-FA_{WC,n}(s)}{FA_{WC,n+1}(s)}\right|\times 100\%.$$ (8)

When both distortion-corrected images are combined into a final image, the difference of final FA maps is caused by the different contribution using two weighting maps as a ratio. Thus, the calculation was performed only in the regions where the difference is large, or the ratio is high. Pixels with a difference in contribution greater than 3 were selected for the calculation, which can be obtained when abs(log_e_(*W*_*f*_(*s*)/*W*_*r*_(*s*)) is greater than abs(log(3)). For example, the ratio between two weighting maps becomes 3 or 1/3 in regions where two adjacent pixels have a shift-difference of half a pixel resulting in 50% overlap in compressed regions. In other words, loss of spatial information has already occurred in such regions and this signal without sufficient spatial information may corrupt further DWI processing and should be discarded. This is achieved with the proposed weighted combination and the SNR in the combined image varies accordingly. Under the assumption that two distortion-corrected images with opposite PE polarity have identical SNR, the change in the SNR map due to the weighted combination can be obtained by using a simple average of two images:

$$SNR{\left(s\right)}_{WC,n}=\frac{1+1/Ratio\;{\left(s\right)}_{WC,n}}{\sqrt{1+1/Ratio{\left(s\right)}_{WC,n}}},$$ (9)

where

$$Ratio{\left(s\right)}_{WC,n}=\exp(\left|\log_{e}(W_{f}{\left(s\right)}^{n}/W_{r}{\left(s\right)}^{n})\right|).$$

The contribution of one of the two images is decreasing when the weighting ratio Ratio(*s*)_*WC,n*_ increases. Therefore, the corresponding SNR map change ranging from 1 (contribution mainly from one locally stretched image) to sqrt(2) (equal contribution from regions with little distortion) is calculated by Eq. 9.

(IV) For comparison, the reversed gradient approaches, which are available in FSL (http://fsl.fmrib.ox.ac.uk/fsl/) via a tool called TOPUP (http://fsl.fmrib.ox.ac.uk/fsl/fslwiki/topup), were applied to *in-vivo* DTI data acquired in the third experiment. After distortion correction was performed with shift maps calculated using algorithms of Jacobian modulation and least-squares restoration, two corresponding final FA maps were calculated.

## Results

**Fig 2:**
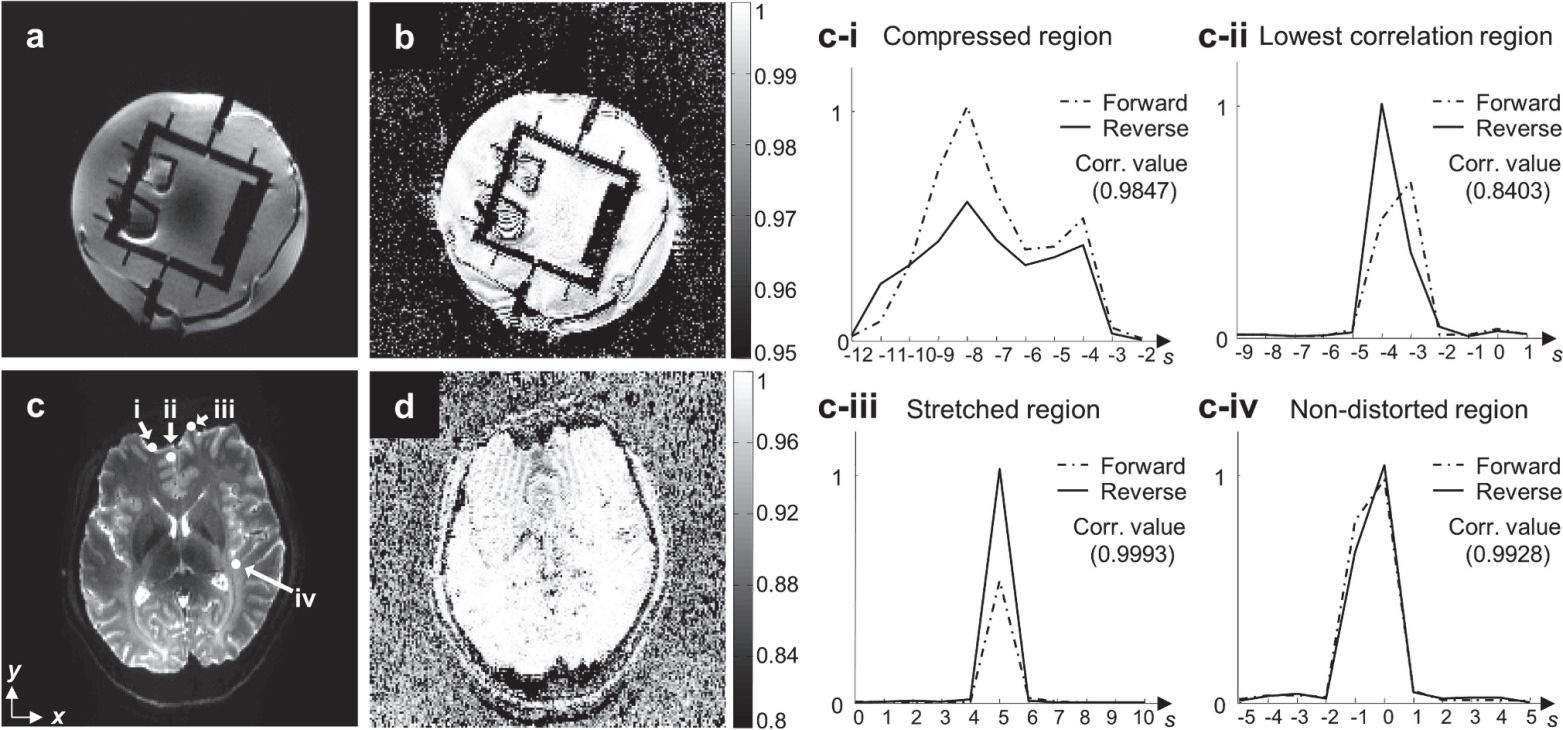
Distorted SE-EPI images of the phantom (a) and human brain (c) and the corresponding correlation maps of the EPI-PSF profiles (b and d). The EPI-PSF profiles in the forward and extended reverse 3D PSF spaces were used for the calculation and collected at four different regions for the intensity comparisons, such as compressed (c-i), lowest correlation (c-ii), stretched (c-iii), and non-distorted regions (c-iv) of the human brain (c) and normalized by the largest value. In these figures, dashed and solid lines stand for forward and reverse PE scheme, respectively. The corresponding cross correlation (Corr.) value between the selected profiles is shown in each figure.

Results of cross correlation between the measured EPI-PSF profiles in the forward and extended reverse PSF spaces are shown in Fig. 2. In the phantom experiment, nearly identical profiles were obtained along the spin-warp PE coordinate (*s*) in the EPI PE coordinates (*y*) from the forward and extended reverse PSF data, as seen from the correlation map in Fig. 2B. Very high cross correlation values (more than 0.95) were calculated in areas with substantial signal. Similar results were obtained from *in-vivo* experiments although the correlation values were slightly reduced to 0.84 in the strongly distorted anterior regions (Fig. 2D and 2C-ii). When the center of mass of the EPI-PSF profiles was calculated along the *s*-direction using a single delta approximation [12], the differences were below 0.5 pixels.

The EPI-PSF profiles in the both PSF spaces were examined at four different regions. Due to compression and stretching, the EPI-PSFs were shifted to negative (Fig. 2C-i and 2C-ii) and positive (Fig. 2C-iii) positions along the *s*-direction from the diagonal line. Only in non-distorted regions, the EPI-PSF was located along the diagonal line (Fig. 2C-iv). Especially in strongly distorted regions (Fig. 2C-i and iii), an intensity difference between the EPI-PSF profiles in the two PSF spaces was clearly seen. Higher and lower intensities were respectively measured in the compressed and stretched regions for the EPI-PSF profiles in the forward 3D PSF spaces and vice versa in the extended reverse 3D PSF spaces. In contrast, the intensities were very similar in the non-distorted region (Fig. 2C-iv) and the difference wasn’t discernible where the field inhomogeneity effects were not strong (Fig. 2C-ii).

**Fig 3:**
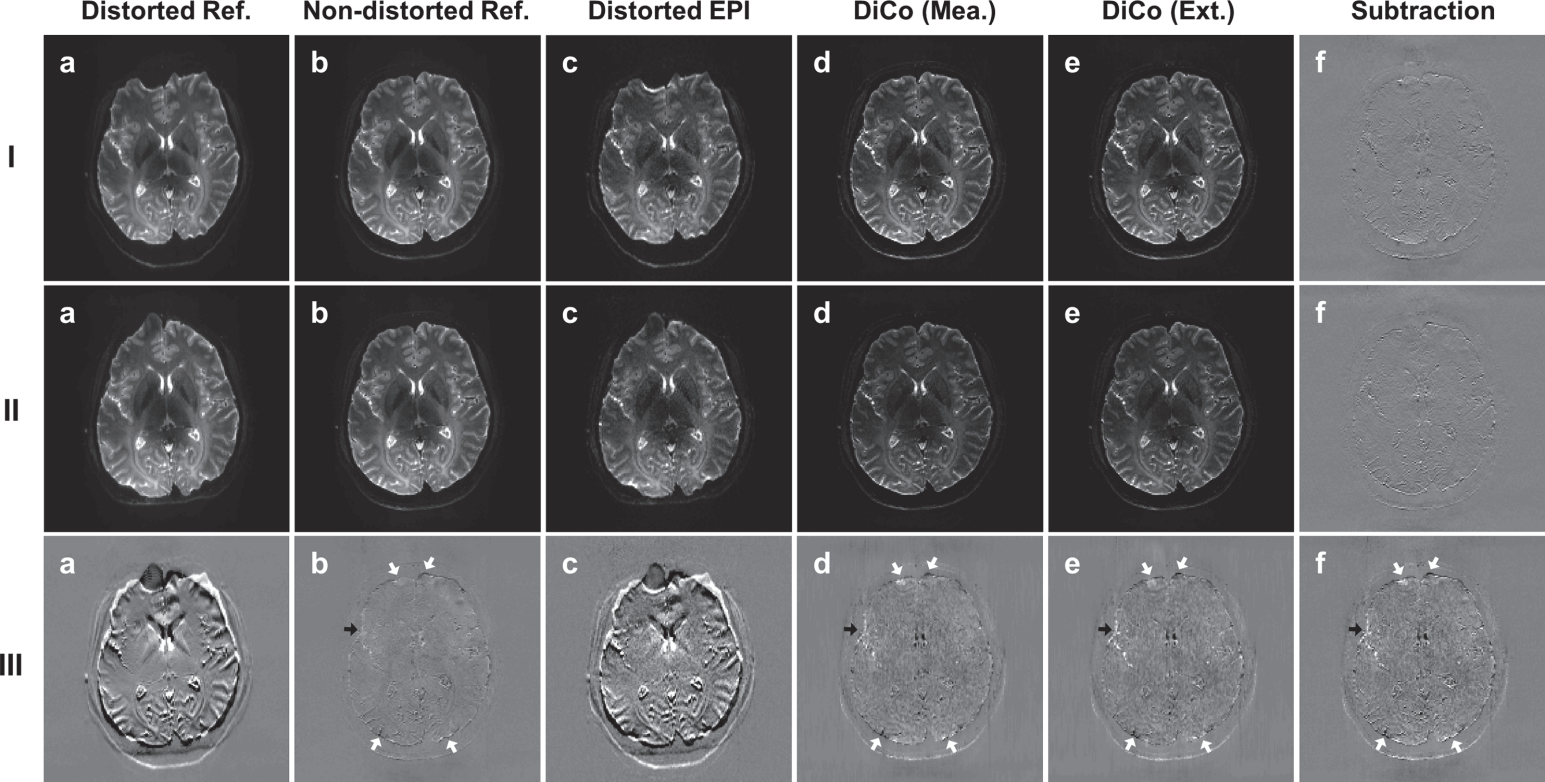
Distortion correction with forward (row I) and reverse (row II) SE-EPIs and their differences (row III): (a and b) distorted and non-distorted reference (Ref.) images calculated from the 3D PSF data, (c) distorted EPI image, (d and e) the distortion-corrected EPI images corrected by the EPI-PSF kernels calculated from the corresponding measured (Mea.) and extended (Ext.) PSF data with forward (row I) and reverse (row II) PE polarity, respectively. (I-f and II-f) subtracted images between (d) and (e), and (III-f) a subtracted image between (I-d) and (II-e), which corresponds to the proof of high quality result obtained by the proposed method.

Fig. 3 shows that the geometrical differences between the forward and reverse SE-EPI images are significantly reduced after the proposed distortion correction. Severe geometric distortions appear near boundary regions of the brain in the forward (Fig. 3I-C) and reverse EPI images (Fig. 3II-C) and the differences are clearly seen in the subtracted image (Fig. 3III-C) due to opposite geometric distortions. In most areas, the differences were reduced after distortion correction. Some residual differences remain in strongly distorted regions (see white arrows) as well as in the ventricle and arterial (see black arrows) regions as shown in Fig. 3III-D,-E, and -F. Interestingly, as shown in the subtracted image (Fig. 3III-B),very similar results were obtained in the non-distorted forward (Fig. 3I-B) and reverse reference image (Fig. 3II-B), which were respectively calculated from the forward and reverse PSF data using Eq. 2.

**Fig 4:**
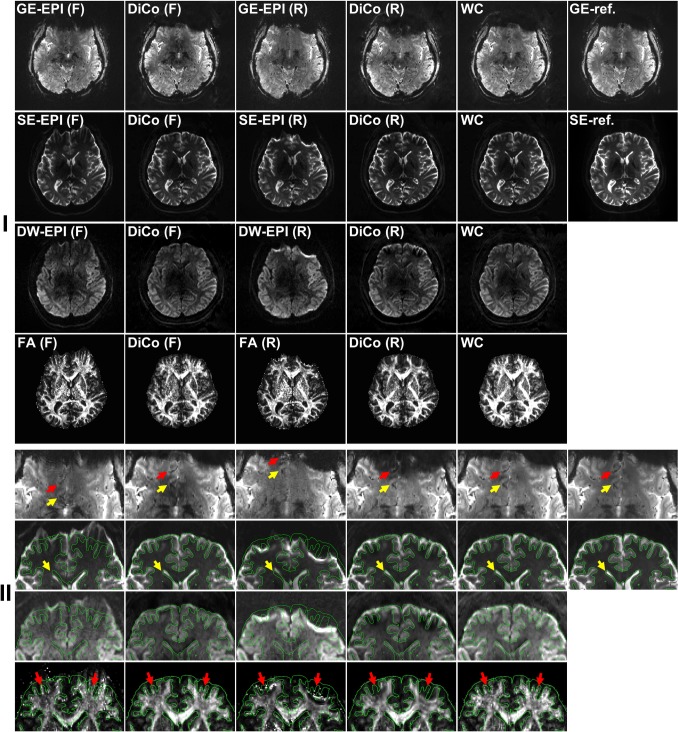
Forward (F) and reverse (R) GE- (I first row), SE- (I second row), and DW-EPIs (I third row) and FA maps (I bottom row) without and with DiCo. An identical slice was chosen for SE- and DW-EPIs and corresponding FA maps. Weighted combination (WC) of the two distortion-corrected images was carried out using the proposed weighting maps (*n* = 2) and a final FA map was calculated based on final SE- and DW-EPIs. Green contours calculated from the SE-reference image (SE-ref.) were overlaid onto the enlarged images (II) of the anterior regions of the full FOV images (I). In the enlarged images of GE-EPIs, two blood vessels in the anterior regions of the GE-reference image (GE-ref) are used as landmarks to verify the correction fidelity.

The original (i.e. distorted) and distortion-corrected forward and reverse GE-, SE-, and DW-EPIs and FA maps are shown in Fig. 4. In forward SE- and corresponding DW-EPI, strongly stretched geometric distortions are visible near boundaries in anterior regions and slightly compressed distortions near the ventricles (see yellow arrows in Fig. 4II second row of enlarged images), and vice versa in the reverse SE- and DW-EPIs, resulting in equivalently distorted FA maps (see red arrows in Fig. 4II bottom row). After the proposed distortion correction, the EPIs and FA maps matched well with the reference image without distortions. In contrast to the SE-EPI pair, very different image intensity appears in the GE-EPI pair, especially near the anterior edge of the brain due to strong signal dropouts in the reverse GE-EPI. Nevertheless, the proposed distortion correction performed very well even in GE-EPIs (see arrows in Fig. 4II first row of enlarged images). Although loss of spatial information due to compressions cannot be recovered by the distortion correction as shown in the distortion-corrected forward and reverse GE-EPIs and FA maps, the proposed weighted combination of EPIs allows generating a final GE-EPI and FA map with efficient spatial information (see WC in Fig. 4I and 4II).

**Fig 5:**
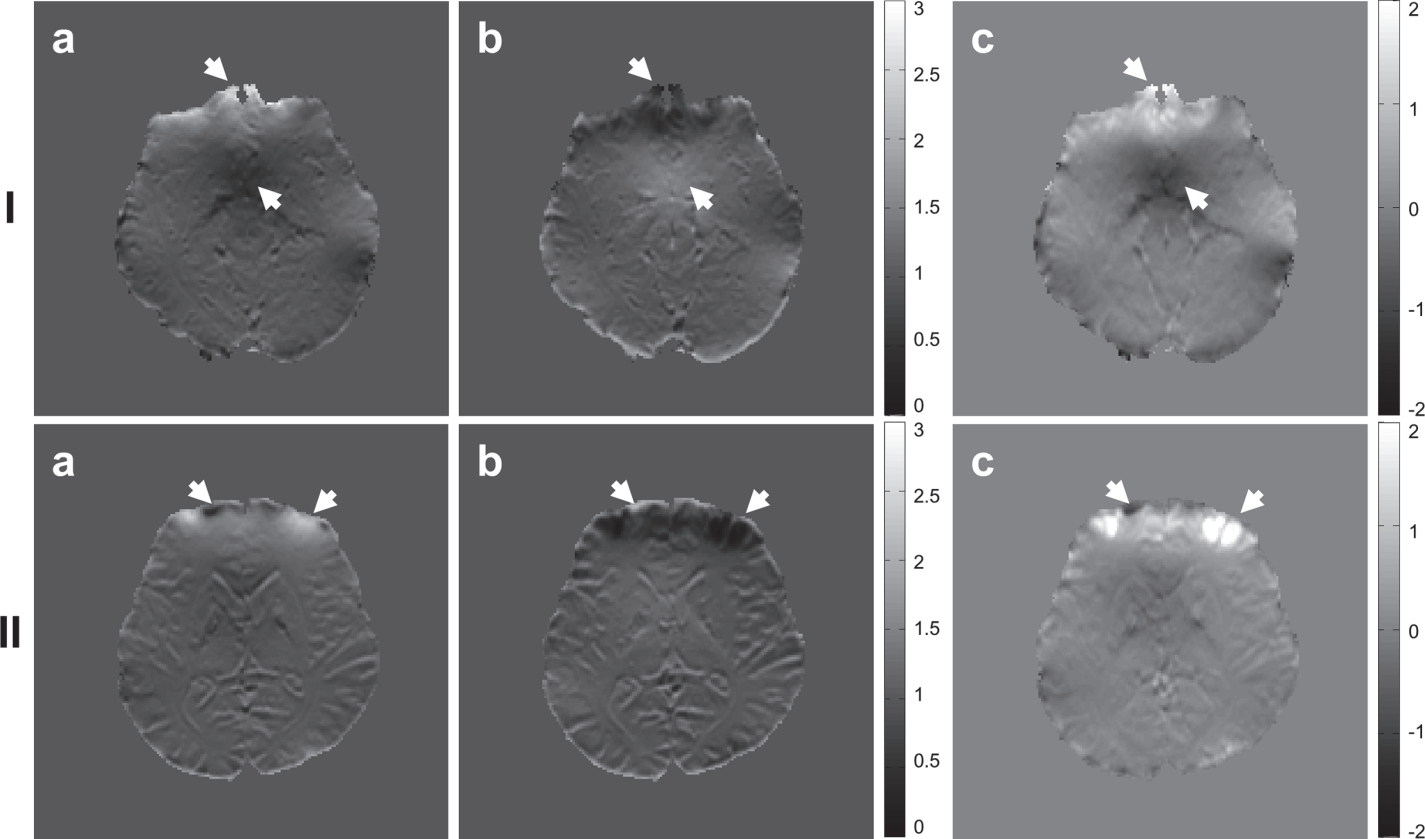
The EPI-PSF kernel based weighting maps and the ratio map in GE- (I) and SE-EPIs (II): two weighting maps, *W*_*f*_(*s*) (a) and *W*_*r*_(*s*) (b), are calculated respectively from the forward and reverse EPI-PSF kernel using Eq. 6 and the ratio (c) is shown in logarithmic scale using the equation log_e_(*W*_*f*_(*s*)/*W*_*r*_(*s*)).

Fig. 5 shows weighting maps calculated from the EPI-PSF kernels. Since the EPI-PSF kernel contains information about both blurring and shift of the PSF due to distortions, the correction procedure considers these effects in calculating the weighting maps. For example, image blurring occurs along the PE direction in the acquired EPI due to the use of a partial Fourier factor of 6/8 together with zero-filling for the image reconstruction. Since the PSF blurring is usually larger near structure boundaries, such as tissue/tissue, tissue/vessels, tissue/ventricles, or tissue/air boundaries in the brain, the locally measured weightings are similar in both maps, as shown in Fig. 5A and 5B. In contrast, weightings due to PSF shifts vary more smoothly (or on global scale) within the slice. Since the protocols were identical with exception of the reversed PE gradient in the EPI measurement, the effects of the PSF shift, but not blurring, are reversed on the weighting maps. Therefore, as shown in the weighting ratio image (Fig. 5C) in logarithmic scale, the effects of PSF shifts are mainly accounted for in the combination process. Compared to the reverse EPI image, the forward EPI image introduces 64 times higher contribution (log_e_8^*n*^ = *n*×2.16, *n* = 2) to the combined image in the anterior regions (see arrows in Fig. 5) and lower contribution near ventricle regions when the combination process is performed.

**Fig 6:**
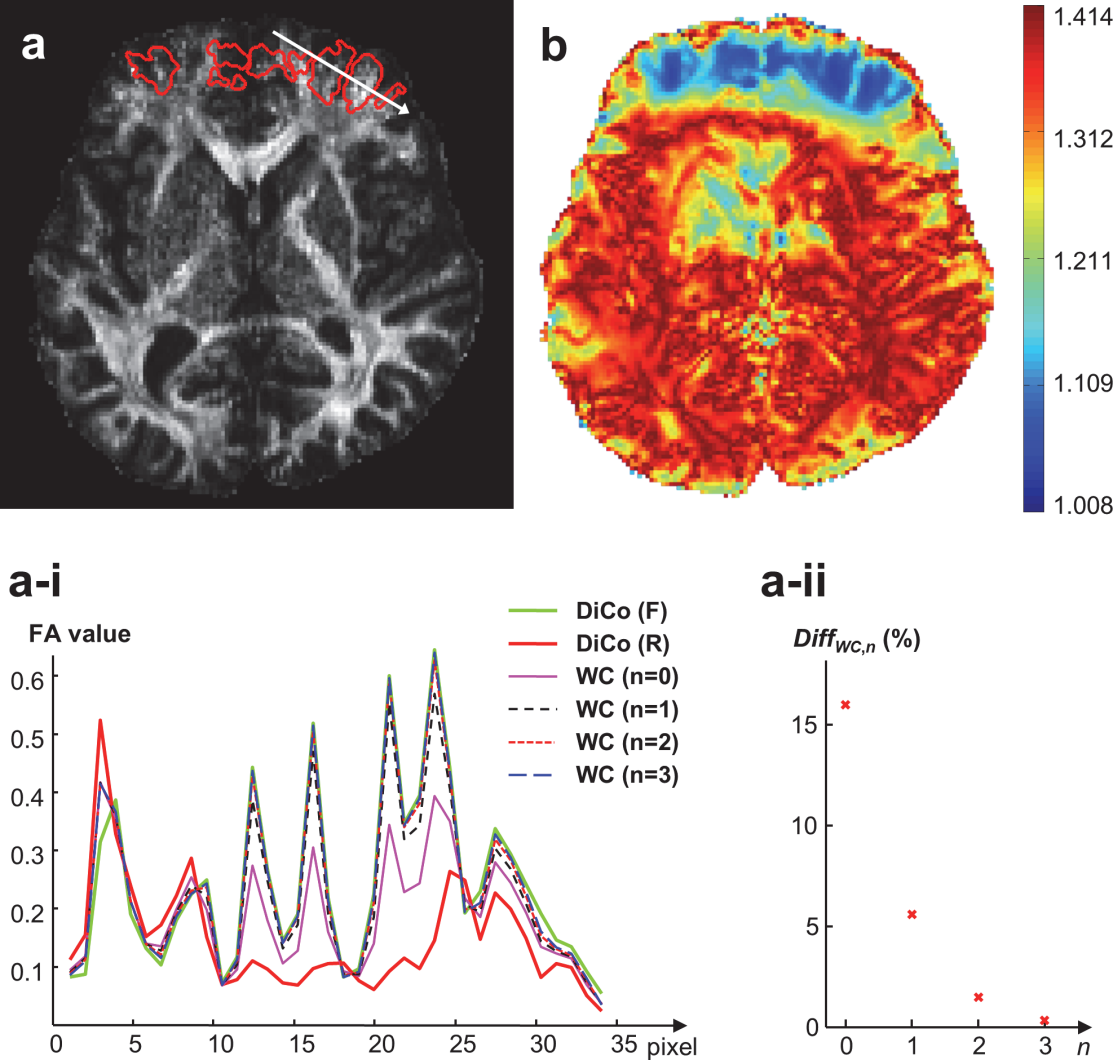
Selection of a suitable weighting exponent *n* for combination. (a) Final FA and (b) corresponding relative SNR map with suitable *n* = 2 gained relative to the source scans; the line profile in (a) is taken along the white arrow; the red-contours mark areas corresponding to exactly same ROIs in where derivatives of pixel shifts are more than 0.5 pixel, (a-i) line profiles obtained from six FA maps, which include forward (F) and reverse (R) FA maps with DiCo and four final FA maps with different weighting exponents (*n* = 0, 1, 2, and 3), (a-ii) mean differences (in %) within red ROIs between five final FA maps with different weighting exponents (*n* = 0, 1, 2, 3, and 4) calculated using Eq. 8. Note that the mean difference of 16% was measured between FA maps obtained by simple (*n* = 0) and weighted averages (*n* = 1), which corresponds to *n* = 0 in (a-ii).

Fig. 6 shows that the proposed weighted summation results in higher effective resolution of the final FA map. As an example, the frontal white matter structure in the reverse distortion-corrected FA map was not resolved due to strong compression (see line profile between pixel 13 and 23 in Fig. 6A-i). The loss of spatial resolution was better recovered with weighted averages (*n*≠0) rather than a simple average (*n* = 0). Under the assumption that the spatial resolution in the stretched areas is more reliable, meaningful FA values from forward images were retained in the final FA map when *n* was 2 or larger. For such values of *n*, the combined map contains signal mainly from one of the two acquisitions in strongly distorted regions with loss of spatial information, as shown in SNR map of the combined image (Fig. 6B). There was only a very small incremental difference (less than 2%) for higher values of *n* (Fig. 6A-ii).

**Fig 7:**
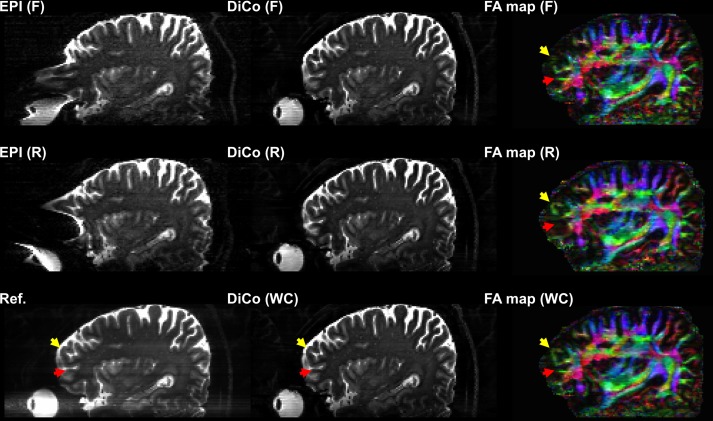
The whole volume of SE-EPI sagittal images without (left) and with DiCo (middle) and corresponding color-coded FA maps (right): forward (F) PE, reverse (R) PE, and the weighted combination (WC) images are compared with the reference (Ref.) without distortions. Yellow and red arrows point on completely recovered anatomical peculiarities in color-coded FA map (WC), but suboptimal recovery in color-coded FA maps (F and R).

Severe geometric distortions vary significantly not only within transverse slices, but also across the slices in a volume. For demonstration purposes, a sagittal reformation of the entire volume (80 slices) was selected, as shown in Fig. 7. Strongly compressed and stretched distortions appear across the slices respectively near upper and bottom boundaries of prefrontal lobe in the forward EPI and vice versa in the reverse EPI. Correspondingly, loss of spatial information occurred in the affected regions of the distortion corrected FA maps (FA map (F) and (R) in Fig. 7) although distortion correction with the proposed method performed very well in both images even in the compressed regions (DiCo (F) and (R) in Fig. 7). Again, the problem can be resolved well by the weighted combination of the two images after the proposed distortion correction, as shown in the final FA map with weighted average (see FA map (WC) in Fig. 7).

**Fig 8:**
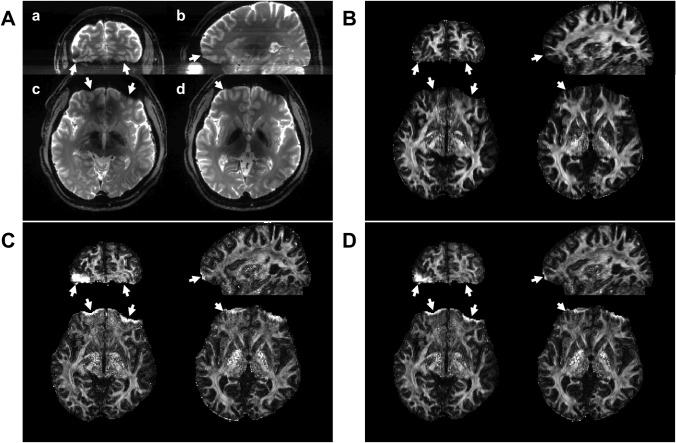
A reference image (A) without distortion, the corresponding final distortion-corrected FA maps with the proposed method (B), the original reversed gradient approaches using algorithms of Jacobian modulation (C) and least-squares restoration (D). For comparison, a coronal (a), sagittal (b) and two axial images (c and d) were selected and white arrows point to the differences between FA maps.

Fig. 8 demonstrates local efficiency of the proposed method in recovering spatial resolution. Although all final FA maps with different distortion correction procedures were overall very similar to each other, improved correction with the proposed method can be found, especially in regions with strong geometric distortions, as shown in Fig. 8 (see arrows). In addition, not only in the affected regions, but also near the connected regions inaccurate distortion calculation occurred with the reversed gradient approaches (see inner parts of anterior regions in axial images in Fig. 8C and 8D). Without such problems, the proposed method allows an improved final FA map (Fig. 8B), which geometrically corresponds to the reference image (Fig. 8A) very well.

## Discussion

In this study, we have proposed and demonstrated an extension of the PSF method for the reversed gradient approach to allow distortion correction in both EPI images with opposite PE directions without an additional PSF reference scan. Combined with further accelerations, the reference scan time for high correction fidelity becomes acceptable. We have further proposed and shown the effectiveness of a novel weighting scheme that accounts for the signal intensity variations using information from the single PSF reference scan. For effective combination of both EPI images, a very precise distortion correction is essential to allow geometrically well-matched forward and reverse EPI images with an anatomical image. In addition, an appropriately weighted combination of corrected images is also necessary to obtain the correct signal levels and exploit information from scans with both PE directions. With the proposed method, it is possible to obtain a distortion-free image using a single PSF scan without loss of spatial resolution. We have shown how the reversed gradient approach can be used to avoid loss of spatial resolution caused by local EPI compression.

An appropriate pair of EPI-PSF kernels for distortion correction can be calculated from a single PSF reference data set by the proposed method. As shown in Fig. 2, a very high cross correlation between the EPI-PSF profiles in both PSF spaces was calculated in phantom and *in-vivo* experiments. This supports our hypothesis that the EPI-PSF profiles in the forward and reverse 3D PSF spaces are identical except for opposite distortions (i.e. mirrored relative to the diagonal line in *s-y* plane). Only in *in-vivo* experiments, slightly reduced correlation values were measured since the EPI-PSF profiles were shifted differently. It seems that the field inhomogeneity was slightly changed due to subject motion between the two PSF reference scans. However, the two EPI-PSF profiles were still very similar, as shown in Fig. 2C-ii. The differences were below 0.5 pixels when counted as a pixel shift although a very high resolution EPI (about 1.2 mm^3^) was acquired in this study. Therefore, the proposed method can correct geometric distortions in both forward and reverse EPIs with sub-voxel accuracy without loss of correction fidelity and even in very high resolution EPI with strong distortions.

Our results emphasize that not only geometric distortions but also intensity distortions of the PSF are caused by the field inhomogeneity. Some residual differences in the subtraction maps between the forward and reverse images still remain in the regions with strong field inhomogeneity or artery and ventricle regions even after distortion correction (see white and black arrows in Fig. 3). Such differences are not likely to be caused by the proposed method since the distortion correction by the measured or extended EPI-PSF kernels produces very small differences in these regions, as shown in Fig. 3I-f and 3II-f. Even in the regions with strong field inhomogeneity (same for regions with arteries and ventricles), very high correlations between the EPI-PSF profiles from the two PSF data were obtained as shown in Fig. 2, which results in identical distortion correction by the proposed method. Nevertheless, the differences were not changed even if either the measured (Fig. 3III-D), extended (Fig. 3III-E), or proposed method (Fig. 3III-F) were used for the distortion correction. These can be explained well with an intensity difference between the EPI-PSF profiles in the two PSF spaces, especially in strongly distorted regions (Fig. 2C-i and iii) and very similar results were obtained even in the subtraction image (Fig. 3III-B) between two non-distorted reference images (Fig. 3I-B and 3II-B) calculated from two PSF reference data with the reversed PE polarity. Therefore, these results show that the difference between the distortion-corrected forward and reverse images seems to be caused mainly in the acquisition, rather than the distortion correction process.

Several sources can cause intensity distortions in DTI data during the acquisition. Although phase dispersion due to the local field inhomogeneity, whether in-plane or through-plane, is recovered at the spin-echo, the echo peak is either blurred or narrowed according to the background gradient sign [7]. Brain-tissue vibrations induced by cardiac pulsation [15,16] can also result in a local echo shift in *k*-space, which leads to signal loss. The vibration artifact manifests itself when partial Fourier imaging is used, as is common in DTI for reducing the TE [17]. Moreover, the high signal variability near the medial parts of the brainstem, cerebellum, and the lateral ventricles in acquired EPI images with *b* = 0 were most likely due to pulsation artifacts [18]. In addition, the relative magnitude of those effects would further increase with T2 decay modulation along the entire readout window and may not be negligible, especially in high resolution EPI at UHF due to longer readout time with faster T2 decay. However, the proposed correction is not affected by the aforementioned problems since only the profile, but not the intensity, of the measured EPI-PSF is used as a kernel for distortion correction.

In severely warped regions, accurate distortion information cannot be calculated only from the SE-EPI pair with opposite distortions. As demonstrated in Fig. 1, geometric distortions occur in EPI acquisition due to shifts of PSF caused by field inhomogeneity. In stretched regions (Fig. 1B), only a PSF exists along the non-distorted (*s*) coordinate in the distorted (*y*) axis and yields a voxel intensity by Eq. 3 in the distorted reference image (Fig. 1A, right), which has identical geometric distortions as in EPI (see distorted reference and EPI in Fig. 3). With the opposite distortion, however, two or more PSFs, which are originally from very different locations, are aligned along the same non-distorted (*s*) coordinate (Fig. 1D) and the summed intensity is acquired in the corresponding EPI with opposite PE polarity. Consequently, one to one intensity matching between the SE-EPI pair is not given to calculate distortion information from the two magnitude images with opposite PE gradient and no unique solution exists in such areas (Fig. 1D). Due to severe wrapping, accurate correction is not possible even near the affected regions (see inner part of anterior regions in Fig. 8C and 8D). In addition, as discussed above, intensity differences between the image pair may further reduce the calculation accuracy. These problems become more obvious at UHF due to increased distortion strength (see arrows in Fig. 8C and 8D). In contrast, the PSF method allows accurate mapping of distortions even in the affected regions and very reliable correction is possible in both images (Fig. 8B).

A similar approach to combine the two distortion-corrected images has been suggested previously by Skare and Bammer [19]. However, it may not be possible to determine the described weighting map, especially in severely warped regions since a smoothed derivative map was calculated from the shift map, which was in turn calculated from the two magnitude images with opposite PE polarity. In addition, strong distortions can lead to a negative value in the derivative map and manual adjustment of the theshold value is requred in these regions [19]. In constrast, the proposed weighting map is calculated from the EPI-PSF kernel in this study. Since the kernel is obtained from the measured PSF data, it produces an accurate modulation function to resolve the signal intensity associated with geometric distortions, which is also assumed in this study as a function of spatial accuracy in the corresponding regions, and doesn’t allow any negative values. Therefore, the proposed weighting allows a more reliable combination. However, other combination methods may be even more efficient.

Loss of local SNR in the combined image is mainly caused by local image compression and thus loss of useful image information and not by the proposed weighted combination. Since strongly compressed distortions cause loss of spatial information, the signal without spatial information content should not be considered and is reduced in the combination. A weighting map is suggested in this study in order to avoid the inclusion of signal without correct spatial encoding. Consequently, the SNR map calculated by Eq. 9 represents the SNR, which can be achieved from two images with opposite PE distortion. Therefore, it improves the final FA map despite unaltered SNR (see Fig. 6A-i and 6B). Without a suitable combination scheme, spatial information is lost leading to potentially erroneous processing results (see unusual high FA values near anterior regions in Fig. 8C and 8D).

In stretched regions, no noticeable accuracy degradation would be expected in calculating the FA value since the distortion patterns are identical across all DW-EPIs and the brain structures geometrically match each other. Although the susceptibility-induced gradient can act as an additional diffusion gradient, which results in an inaccurate FA value, its strength is usually much smaller than the applied diffusion gradient strength. In addition, it is partially compensated for by the use of the logarithm of the ratio of the DW to non-DW intensity, in which case the effect of the local susceptibility-induced gradient is cancelled [20]. Therefore, the lost spatial information due to compression can be resolved from the corresponding EPI image with opposite distortions (Fig. 7).

We empirically demonstrated that a suitably weighted combination rather than simple average of both distortion-corrected images can allow a final FA map with effective spatial resolution in strongly distorted regions (Fig. 6). When final FA maps with and without weighted combination were compared, the mean difference in moderately warped frontal white matter regions was at least 16%. While this is done for selected regions, our results demonstrate that the accuracy of diffusivity parameters can be improved substantially with a suitably weighted average.

The proposed method can be generalized to other EPI-based studies, such as fMRI or perfusion MRI (pMRI), which are usually performed with GE-EPI. As shown in the GE-reference image (Fig. 4) calculated from GE-PSF data, the PSF-based method allows mapping of the PSF with identical protocols corresponding to GE-EPI even in regions with strong signal dropouts and the proposed method is compatible with a reversed gradient GE-EPI approach with high correction fidelity. A final image, which is almost identical to the GE-reference image, was obtained with the proposed weighted combination, originally designed for SE-EPI. However, the combination of reversed gradient GE-EPI data requires more careful attention than reversed gradient SE-EPI. As discussed in a previous study [21], the effective TE of gradient-echo EPI varies spatially with local susceptibility-induced gradients and the patterns will be reversed with the opposite PE gradient. Signal loss from through-slice phase variation (slice dropout artifact) specific to GE-EPI further complicates the situation. Therefore, a new framework combining forward and reverse phase-encoded GE-EPIs needs to be further investigated in order to consider the spatially-dependent TE variations as well as to maximize the functional information. The currently proposed combination will lead to an effective weighting between the shortened and prolonged effective TE of both PE polarities and thus in part mitigate the effect.

Compared to the previous study [14], a larger kernel size of up to 13 instead of 6 was used in this study to cover the complete EPI-PSF profile, especially in strongly compressed areas (Fig. 2C-i). Several PSFs originating from different regions can be shifted along the y-directions in the s-coordinates due to compression and collapse to the same y-coordinate. The entire PSFs become an EPI-PSF profile and thus the width is increased since it is collected along the s-direction in the y-axis. Since the complete information is measured as the EPI-PSF kernel, the b = 0 images are fully corrected by the EPI-PSF kernel even in severely compressed regions, as shown in Figs. 4 and 5. If the EPI-PSF profile is not covered completely, information about distortions is lost in the kernel leading to suboptimal correction. However, an increase of kernel size could introduce noise into the kernel and generally causes blurring of the EPI-PSF profile, which results in a blurred FA map. Therefore, a flexible kernel size based on the width of the EPI-PSF profile or an additional denoising procedure will be considered in future for further improvements.

The additional PSF reference scan time becomes a main disadvantage of this method compared to the reversed gradient approaches [4,5,6,7,8] since it sacrifices some potential SNR in EPI acquisitions. Nevertheless, the extra scan time may be necessary in order to provide high spatial fidelity as well as correction robustness. Since the profile measurement is usually performed without diffusion gradient and the PSF data has high SNR, the correction is very robust against noise. In addition, SNR is further increased with averaging effects caused by the multi-shot nature of the PSF sequence. Since identical geometric distortions can be guaranteed with the opposite PE gradient, as demonstrated in Figs. 2 and 3, the proposed method can yield robust correction even in DW-EPIs with low SNR.

The work in this study assumes the B0 field homogeneity does not change during subsequent EPI acquisitions. Inaccurate correction may occur when subject motion alters the magnetic field distribution relative to the object and thus alter the geometric distortions. Image based coregistration can reduce such errors for magnetic field changes that move with the object. In order to fully account for motion-induced field changes, the proposed approach should be applied together with a real-time motion detection and motion-induced varying distortion correction [22,23].

Loss of spatial information in the FA map cannot be avoided if either the forward or reverse PE DW-EPIs are used for the calculation since the compressed distortions usually appear within a slice (Fig. 4) as well as in a volume (Fig. 7). Even with very accurate distortion correction, calculating only either the forward or reverse FA maps may not provide sufficient accuracy when anatomical connectivity from one cortical area to another is studied in the human brain using diffusion tractography [24,25,26], especially near pre-frontal (see SE-EPI (F) and (R) in Fig. 7) or temporal areas where severe geometric distortions usually appear. Therefore, the proposed method for the reversed gradient approach can be a good choice to provide distortion-free DW images and to utilize the distortion-free information for such studies.

## Conclusion

We introduce an extension of the PSF method for reversed gradient approaches to calculate a distortion-free image for EPI-based application studies without bias due to the PE direction. Since the proposed method extends the PSF reconstruction and yields an additional PSF dataset containing opposite distortions equivalent to a reversed gradient PSF data set, both geometric distortions in the forward and reverse EPI images can be corrected from a single PSF reference scan. Furthermore, the weighting maps for an appropriate combination of the two images were derived from the corresponding EPI-PSF kernels. The results show that very accurate geometric distortion correction in both images can be carried out by the proposed method and that the proposed summation of the two distortion-corrected images can resolve the local loss of spatial information due to compression. The benefits of the proposed method for reversed gradient approaches are demonstrated in phantom and *in-vivo* at 7 Tesla.
